# Vibrational Force on Accelerating Orthodontic Tooth Movement: A Systematic Review and Meta-Analysis

**DOI:** 10.1055/s-0042-1758070

**Published:** 2022-12-13

**Authors:** Amin Akbari, Vaibhav Gandhi, Jie Chen, Hakan Turkkahraman, Sumit Yadav

**Affiliations:** 1 Department of Mechanical and Energy Engineering, Indiana University–Purdue University Indianapolis, Indianapolis, Indiana, United States; 2 Division of Orthodontics, University of Louisville School of Dentistry, Louisville, Kentucky, United States; 3 Department of Orthodontics and Oral Facial Genetics, Indiana University School of Dentistry, Indianapolis, Indiana, United States; 4 Division of Orthodontics, School of Dentistry, University of Connecticut Health, Farmington, Connecticut, United States

**Keywords:** accelerated orthodontic tooth movement, systematic review, vibrational force

## Abstract

This study aimed to systematically gather and analyze the current level of evidence for the effectiveness of the vibrational force in accelerating orthodontic tooth movement (OTM). This systematic review was conducted using three electronic databases: Scopus, PubMed, and Google Scholar until March 2022. The search was done through the following journals: *European Journal of Orthodontics* , *American Journal of Orthodontics and Dentofacial Orthopedics* , *The Angle Orthodontist* , *Progress in Orthodontics* , and *Seminars in Orthodontics* . Human or animal studies that have evaluated the effect of vibrational force on the rate of OTM were selected. A meta-analysis was performed for the rate of canine movement per month. Database research, elimination of duplicate studies, data extraction, and risk of bias assessment were performed by authors independently and in duplication. A fixed and random-effect meta-analysis was performed to evaluate the effect of vibrational forces. A total of 19 studies (6 animal and 13 human studies) that met the inclusion criteria were included. Meta-analysis was performed based on four human clinical trials. Three out of four studies showed no significant difference in the rate of canine movement between vibrational force and control groups. The limitation of this study was the small sample size and significant heterogeneity among the studies. Although vibrational forces have been shown to accelerate OTM in experimental studies, the results are inconsistent in clinical studies. The inability to apply desired peak load to the targeted teeth may be the main factor in inconsistent clinical outcomes.

## Introduction

Orthodontic treatment with fixed appliances normally takes 20 to 30 months. 1 2 3 Prolonged treatment duration may lead to patient compliance “burnout” as well as iatrogenic damage like root resorption, gingival enlargement, white spot lesions, and dental caries. 4 5 6 7 Therefore, decreasing the total treatment duration by accelerating the tooth movement has been of interest to both patients and clinicians. Several innovative methods have been attempted to accelerate the rate of orthodontic tooth movement (OTM) including biological, biomechanical, physical, and surgical approaches. 1 8 9 Surgical methods, such as alveolar corticotomy, accelerate tooth movement by inducing regional inflammation causing increased bone modeling and remodeling. 10 11 However, patients are less receptive to these methods due to the invasiveness and increased treatment cost. Recently, nonsurgical techniques like low-level laser therapy, pulsed electromagnetic fields, and the mechanical vibration are gaining more interest and are preferred over the invasive methods for accelerating OTM. 12 13 14

The vibrational force (VF) has been proven to have an anabolic effect on bone. 15 16 17 In addition to an osteogenic effect, a VF has also anti-osteoclastic effects. 15 18 19 In orthodontics, a VF increases osteoblasts/osteoclasts activities resulting in higher bone modeling and remodeling, 20 21 and it can also accelerate tooth movement by activating nuclear factor kappa B signals in osteoblasts that can increase bone metabolism as a result of cellular interactions in osteoclasts, osteoblasts, and osteocytes. 22 OTM is accomplished by bone resorption on the compression side as a result of increased osteoclast activity and bone formation on the tension side due to elevated osteoblast activity. Additionally, the application of VF on a tooth further increases the aggregated cell activity in the corresponding periodontal ligament (PDL), 23 thus, accelerating the basal metabolic rate in the alveolar bone (a key requirement for accelerated tooth movement [ATM]). 20 23 Studies in rats show that VF results in significantly faster OTM. 14 24 25

Clinical studies have been conducted to evaluate the effect of commercially available vibrating devices on the rate of tooth movement, however, the results are inconsistent. 26 27 28 Understanding the reason for the inconsistency is critical for translating the technology to clinical treatment.

Like medicine, an effective VF stimulation needs to be delivered to generate the expected clinical outcome. The VF level is determined primarily by factors such as peak load (PL), vibrational frequency, application frequency (Af), and application duration (AD). PL should be used to represent intensity rather than the probe's displacement or acceleration as reported in prior work because cells sense stress changes, which are directly linked to PL. 24 25 29 The effective PL or its associated stress should be within a range, called dose, while the PL outside the range has either no effects due to insufficient stimulation or damage because of overstimulation. To ensure an effective and consistent OTM, the VF device must be able to deliver the specified dose to the tooth intended to be moved.

Thus, a comprehensive review of the efficacy of VF on accelerating OTM in both animals and humans is still needed. Animal studies have better control of the level of stimulation, which can be used to prove the VF treatment's efficacy. The level of stimulation on the individual tooth in the clinical studies is challenging to control, which may result in inconsistent outcomes. The dominant factor that causes the inconsistency needs to be identified.

### Objective

This systematic review and meta-analysis were undertaken with the aim to evaluate the causes of the inconsistent efficacy of the intraoral vibration devices on the rate of OTM in the clinic.

## Methods

### Protocol

Institutional review board submission and approval were not required for this study. The present systematic review is conducted according to the guidelines of the Cochrane Handbook for Systematic Reviews of Interventions version 5.1.0, 30 and is reported according to the Preferred Reporting Items for Systematic Reviews and Meta-Analyses (PRISMA) statement. 31 The animal component of this systematic review reported based on the Animal Research Reporting In Vivo Experiment (ARRIVE) guidelines. 32

### Eligibility Criteria

The criteria for considering studies for this review (PICOS) were the following: (1) Participants: orthodontic patients or laboratory animals to access OTM; (2) Intervention: application of VFs; (3) Comparison: pretreatment versus posttreatment/postintervention changes; (4) Outcome: rate of tooth movement; (5) Study design: randomized clinical trials (RCTs), prospective or retrospective radiometric studies, animal studies.

### Information Sources and Search Strategy

This review was conducted using three electronic databases: Scopus, PubMed, and Google Scholar until November 2021. The following search strategy was developed for databases in order to retrieve records using the English language and controlled vocabulary (when available) relating to accelerated orthodontics using VFs: Step 1 (“Vibrational stimulation” OR “resonance vibration” OR “vibration”), Step 2 (“Rapid” OR “acceleration” OR “speed” OR “rate” OR “short”), Step 3 (“Tooth movement” OR “tooth retraction”), and Step 4 (“Orthodontics” OR “orthodontic”). The search was done in English language only through the following journals: *European Journal of Orthodontics* , *American Journal of Orthodontics and Dentofacial Orthopedics* , *The Angle Orthodontist* , *Progress in Orthodontics* , and *Seminars in Orthodontics* . In addition, the literature review was limited to those studies that used the following commercial vibrational devices: AcceleDent (OrthoAccel Technologies Inc., Houston, Texas, United States), VPro5 (Propel Orthodontics, Milpitas, California, United States), Tooth Masseuse (no longer available), and Electronic toothbrush (Oral-B Triumph, OD17; Procter & Gamble, Cincinnati, Ohio, United States) ( Supplementary File S1 ).

### Study Selection

Both human and animal studies were considered for this systematic review. Inclusion criteria were RCTs or non-RCTs, prospective studies, and animal studies. We included studies that investigated the effect of VFs on the OTM. Exclusion criteria were meta-analysis or systematic reviews, review articles, retrospective studies, abstracts, letters from the editor, opinion articles, case reports, and case series.

### Data Items and Collection Form

A customized data collection form was created and used to gather information from the selected studies. This information included authors, year of publication, type of studies, details of the interventions, characteristics of participants, duration of treatment, and outcome measures ( Tables 1 and 2 ). The data extraction was performed by authors (A.A./G.V.) independently and in duplication. An attempt to contact the authors was made for any missing information. In case of disagreement, a third reviewer (Y.S.) was contacted to provide an independent decision on the conflict.

**Table 1:** Summary of the included animal studies

Study	Population	Intervention (vibration parameters)				Observation (OTM)	Result
		PL/PA	Vf (Hz)	AD (min)	Af		
Nishimura et al (2008) 24	42 Wistar rats (male, 6 wk, 150 g)	1 m/s ^2^	60	8	Once a week	Maxillary first molar	The extent of tooth movement on day 21 was significantly greater by 15% in the Resonance vibration group than in the Control group
AlSayagh (2014) 35	14 Albino rabbits (male, 1,400 g)	–	113	10	3 times a week	Mandibular central incisors	The amount of tooth movement was greater in the vibration group when compared with the control group on all days but the only significant differences were shown in the amount of tooth movement at incisal edge on 10th, 19th, and 22nd days
Kalajzic et al (2014) 36	26 Sprague-Dawley rats (female, 6 wk)	40 cN	30	10	Twice a week	Maxillary first molar	Orthodontic alone group: 0.486 ± 0.178 mm Orthodontic + vibration group: 0.242 ± 0.139 mm
Yadav et al (2015) 21	64 CD1 mice (male, 12 wk, 24–30 g)	1 cN	5	15	Every 3 days	Maxillary first molar	There was no difference in the rate of tooth movement between the different experimental groups. However, the maximum tooth movement was observed in the spring + 10 Hz group, and the least was in the spring + 20 Hz group
			10				
			20				
Takano-Yamamoto et al (2017) 14	70 Wistar rats (male, 25 wk, 410 g)	3 gf	70	3	Once a week	Maxillary first molars	The most effective magnitude of vibration to accelerate orthodontic tooth movement of the maxillary molar of adult rats was 3 gf at 70 Hz for 3 minutes per week
		1 gf	58				
		50 gf	268				
Alikhani et al (2018) 25	206 Sprague-Dawley rats (adult, male, 120 d, 400 g)	0.5 m/s ^2^	30	5 to 10	Daily	Maxillary first molars	Application of vibration at day 14 significantly increased the rate of tooth movement 2.4-fold (10 cN group) and 2.5-fold (25 cN group). At 28 days vibration caused a 2.3- and 2.4-fold increase in rate of tooth movement in 10 cN and 25 cN groups, respectively
			60				
			120				

Abbreviations: AD, application duration; Af, application frequency; OTM, orthodontic tooth movement; PA, probe's acceleration; PL, peak load; Vf, vibrational frequency.

**Table 2:** Summary of the included human studies

Study	Participants	Type of device	Intervention (vibration parameters)				Observation (OTM)	Result	Study design
			PL (cN)	Vf (Hz)	AD (min)	Af			
Kau et al (2010) 37	14 patients (mean age: 20.3y) (age range 12.1 to 56.6y)	AcceleDent Type 1	20	30	20	Daily	Canine retraction	The total rate of movement: Mandible: 0.526 mm/week or 2.1 mm/28-day month. Maxillary: 0.759 mm/week or 3.0 mm/28-day month	Prospective clinical study
Miles et al (2012) 38	66 consecutive patients (E: age- 13.1, M- 14, F- 19) (C: Age- 13, M- 12, F- 21)	Tooth Masseuse	6	111	20	Daily	Lower anterior alignment	No advantage in using the Tooth Masseuse for 20 minutes per day for the early resolution of crowding	RCT
Pavlin et al (2015) 39	45 patients (E- 21 patients, C: 18 patients)	AcceleDent	25	30	20	Daily	Canine retraction	The average monthly rate of tooth movement: AcceleDent group: 1.16 mm/month (95% CI: 0.86–1.46). Control group: 0.79 mm/month (95% CI: 0.49–1.09). The mean difference: 0.37 mm/month (95% CI: 0.07–0.81). 48.1 ± 7.1% faster movement in AcceleDent group	RCT
Woodhouse et al (2015) 40	81 patients (Accel: *n* = 29, age- 13.9) (Sham: *n* = 25, age- 14.1) (Control: *n* = 27, age- 14.4)	AcceleDent	25	30	20	Daily	Lower anterior alignment	No significant differences among groups in time to reach initial or final alignment	RCT
Leethanakul et al (2016) 41	15 patients (11 females, 4 males)	Electronic Brush	25	125	5	3/day	Canine retraction	T0-T1: Experiment- 0.63 ± 0.06 mm, Control- 0.63 ± 0.06 mm ( *p* = 1.000) T0-T3: Experiment- 2.85 ± 0.17 mm, Control- 1.77 ± 0.11 mm ( *p* < 0.001)	Split-mouth RCT
Miles et al (2016) 42	40 patients (E: Age- 12.7, M- 6, F- 14) (C: Age- 13, M- 8, F- 12)	AcceleDent Aura	25	30	20	Daily	Lower anterior alignment	Little's irregularity index (median): Base line: AcceleDent- 3.9 mm, Control- 4.4 mm ( *p* = 0.46) 10 weeks: AcceleDent- 1.7 mm, Control- 1.5 mm ( *p* = 0.65)	RCT
Liao et al (2017) 43	13 patients (age: 13.6y; mean: 12.1 to 15.5y)	Single tooth Vibration	20	50	10	Daily	Canine retraction	Total space closure Vibration group: 4.27 mm (SD: 0.72). Control group: 3.5 mm (SD: 0.64)	Split-mouth RCT
Miles et al (2018) 44	40 patients (E: age- 12.7, M- 6, F- 14) (C: age- 13, M- 8, F- 12)	AcceleDent Aura	25	30	20	Daily	Canine retraction	The average monthly rate of tooth movement: AcceleDent group: Right- 1.41 ± 0.5, Left- 1.25 ± 0.51. Control group: Right- 1.30 ± 0.61, Left- 1.25 ± 0.46. No difference of the rate of extraction space closure	RCT
Alansari et al (2018) 45	75 patients Control: *n* = 13, age- 30.8, M- 5, F- 8 7 Sham: *n* = 13, age- 28.6, M- 4, F- 9 7 HFA: *n* = 14, age- 31.8, M- 6, F- 8 5 Sham: *n* = 5, age- 24.7, M- 2, F- 3 5 HFA: *n* = 13, age- 29.9, M- 7, F- 6	VPros5	30	120	5	Daily	Invisalign tracking	Vibration stimulation using VPro5 for 5 minutes a day can reduce the interval between aligner change without affecting the efficiency of treatment	RCT
Katchooi et al (2018) 46	27 patients (E: age- 31.4, M- 6, F- 7) (C: age- 34.6, M- 6, F- 7)	AcceleDent	25	30	20	Daily	Incisal irregularity	Change in the incisal irregularity: Maxilla: E = 3.56 ± 0.9 mm, C = 3.36 ± 1.8 mm. Mandible: E = 3.56 ± 2.2 mm, C = 3.74 ± 2.1 mm. The AcceleDent Aura device had no effect on the end-of-treatment alignment achieved with a 1-week Invisalign change regimen	RCT
DiBiase et al (2018) 47	81 patients (Accel: *n* = 22, age- 13.6) (Sham: *n* = 19, age- 13.9) (Control: *n* = 20, age- 14.3)	AcceleDent	0.2N	30	–	Daily	Mandibular canine retraction	No benefits of vibration in terms of mandibular space-closure rate, treatment duration, and final treatment outcome	RCT
Taha et al (2020) 48	22 patients (E: *n* = 10, age- 15.9, M- 3, F- 7) (C: *n* = 11, age- 15.09, M- 4, F- 7)	AcceleDent Aura	25	30	20	Daily	Canine retraction	Amount of tooth movement: T1: E = 1.39 ± 0.36 mm, C = 1.12 ± 0.22 mm ( *p* = 0.058). T2: E = 2.49 ± 0.76 mm, C = 2.59 ± 0.38 mm ( *p* = 0.702). T3: E = 3.37 ± 1.39 mm, C = 3.54 ± 0.24 mm ( *p* = 0.706). No significant differences for the rate of canine retraction	RCT
Reiss et al (2020) 49	40 patients (E: age- 31.4, M- 10, F- 10) (C: age- 34.6, M- 10, F- 10)	AcceleDent	–	–	–	Daily	Lower anterior alignment	Irregularity index T0: E = 7.24 (5.68, 8.59), C = 8.96 (6.19, 11.21) ( *p* = 0.792). T1: E = 4.26 (3.11, 5.3), C = 5.24 (4.01, 7.22) ( *p* = 0.675). T2: E = 2.33 (1.92, 3), C = 2.96 (2.23, 3.8) ( *p* = 0.442). T3: E = 0.97 (0.69, 1.54), C = 1.22 (1.01, 1.75)	RCT

Abbreviations: AD, application duration; Af, application frequency; CI, confidence interval; HFA, high-frequency acceleration; OTM, orthodontic tooth movement; PL, peak load; RCT, randomized clinical trial; SD, standard deviation; Vf, vibrational frequency.

### Risk of Bias and Quality Assessment of Individual Studies

After imposing exclusion and inclusion criteria, several RCTs or non-RCTs, cohort studies, and animal studies addressing our PICO question were found. The Systematic Review Center for Laboratory animal Experimentation (SYRCLE) tool and ARRIVE criteria were used to assess the risk of bias (RoB). 33 This tool has been specifically designed to assess the RoB of animal intervention studies, and it consists of 10 items related to selection bias, performance bias, detection bias, friction bias, report bias, and other biases. The RoB attributed to each domain could be high, unclear, or low ( Tables 3 and 4 ).

**Table 3:** Risk of bias assessment for animal studies using SYRCLE's risk of bias tool

Item	Type of bias	Domain	Review authors judgment	Nishimura et al (2008)	AlSayagh (2014)	Kalajzic et al (2014)	Yadav et al (2015)	Takano-Yamamoto et al (2017)	Alikhani et al (2018)
1	Selection bias	Sequence generation	1	No	Unclear	No	Unclear	Unclear	No
2	Selection bias	Baseline characteristics	2	Yes	Yes	Yes	Yes	Yes	Yes
3	Selection bias	Allocation concealment	3	No	Unclear	No	Unclear	No	No
4	Performance bias	Random housing	4	Unclear	Unclear	No	No	No	No
5	Performance bias	Blinding	5	No	No	No	No	No	No
6	Detection bias	Random outcome assessment	6	No	No	No	No	No	No
7	Detection bias	Blinding	7	No	No	No	No	No	No
8	Attrition bias	Incomplete outcome data	8	Yes	Yes	Yes	Yes	Yes	Yes
9	Reporting bias	Selective outcome reporting	9	Yes	Yes	Yes	Yes	Yes	Yes
10	Other	Other sources of bias	10	Unclear	Unclear	Unclear	Unclear	Unclear	Unclear

Abbreviation: SYRCLE, Systematic Review Center for Laboratory animal Experimentation.

Note: Review Authors Judgment: (1) Was the allocation sequence adequately generated and applied?; (2) Were the groups similar at baseline or were they adjusted for confounders in the analysis?; (3) Was the allocation adequately concealed?; (4) Were the animals randomly housed during the experiment?; (5) Were the caregivers and/or investigators blinded from knowledge which intervention each animal received during the experiment?; (6) Were animals selected at random for outcome assessment?; (7) Was the outcome assessor blinded?; (8) Were incomplete outcome data adequately addressed?; (9) Are reports of the study free of selective outcome reporting?; (10) Was the study apparently free of other problems that could result in high risk of bias?

**Table 4:** Quality assessment of included studies in reference to the Animal Research Reporting In Vivo Experiment (ARRIVE) guidelines

ARRIVE criteria	Nishimura et al	AlSayagh	Kalajzic et al	Yadav et al	Takano-Yamamoto et al	Alikhani et al	Total %
Title	1	1	1	1	1	1	100.0
Abstract	1	1	1	1	1	1	100.0
Background information	1	1	1	1	1	1	100.0
Primary and secondary objectives	1	1	1	1	1	1	100.0
Ethical statement	1	1	1	1	0	1	83.3
Study design, allocation concealment, blinding, and randomization	0	0	0	0	0	0	0.0
Experimental procedure with precise details	1	1	1	1	1	1	100.0
Experimental animal details including species, gender, age, weight, and source	1	1	0	1	1	1	83.3
Housing and husbandry conditions such as type of cage, light/dark cycle, temperature, access to food and water	1	1	0	0	1	0	50.0
Sample size	1	1	1	1	1	1	100.0
Allocation of animals to experimental groups, randomization	0	1	0	1	1	0	50.0
Experiment outcomes	1	1	1	1	1	1	100.0
Statistical analysis	1	1	1	1	1	1	100.0
Baseline data, health status of animals	1	1	0	1	1	1	83.3
Number of animals analyzed or reasons for exclusion	1	1	1	1	1	1	100.0
Outcomes and estimation, results for each analysis	1	1	1	1	1	1	100.0
Adverse events	0	0	0	0	0	0	0.0
Interpretation, scientific implications, study limitations	1	0	0	1	1	1	66.7
Generalizability and translation, relevance to human biology	0	0	1	1	0	0	33.3
Funding sources, conflict of interest	0	0	0	0	1	1	33.3
Total	15	15	12	16	16	15	

Note: 1 = present, 0 = absent.

The methodological index for nonrandomized studies (MINORS) was utilized to assess the RoB of human studies ( Table 5 ). 34 We chose to include eight events for noncomparative studies and 12 events for the comparative studies in the RoB assessment and a higher event rate allows giving a more precise estimate of the influence of studied determinants. The items were scored 0 if not reported; 1 when reported but inadequate; and 2 when reported and adequate. The global ideal score was 16 for noncomparative studies and 24 for comparative studies. To ascertain the validity of eligible human studies, pairs of reviewers (A.A./G.V.) working independently and with adequate reliability determined the accuracy of the objectives, adequacy of concealment of allocation, blinding of patients, data collectors, outcome assessment, the extent of loss to follow-up (i.e., proportion of patients in whom the investigators were not able to ascertain outcomes), and prospective calculation of the study size.

**Table 5:** Risk of bias assessment for human studies using MINORS risk of bias tool

Criteria	1	2	3	4	5	6	7	8	9	10	11	12	Total
Kau et al (2010)	2	2	2	2	0	2	0	0					10
Miles et al (2012)	2	2	2	2	2	2	2	2	2	2	2	2	24
Pavlin et al (2015)	2	2	2	2	2	2	1	2	2	2	1	2	22
Woodhouse et al (2015)	2	2	2	2	2	2	2	2	2	2	2	2	24
Leethanakul et al (2016)	2	0	0	2	2	2	2	0	2	2	2	2	18
Miles et al (2016)	2	2	2	2	2	2	2	2	2	2	2	2	24
Liao et al (2017)	2	2	2	2	0	2	1	0	2	2	2	2	19
Miles et al (2018)	2	2	2	2	2	2	2	2	2	2	2	2	24
Alansari et al (2018)	2	2	2	2	2	2	0	2	2	2	2	2	22
Katchooi et al (2018)	2	2	2	2	2	2	2	2	2	2	2	2	24
DiBiase et al (2018)	2	2	2	2	2	2	2	0	2	2	2	2	22
Taha et al (2020)	2	2	2	2	1	2	2	2	2	2	2	2	23
Reiss et al (2020)	2	2	2	2	2	2	2	2	2	2	2	2	24

Abbreviation: MINORS, methodological index for nonrandomized studies.

Note: Criteria: (1) A clearly stated aim; (2) Inclusion of consecutive patients; (3) Prospective collection of data; (4) Endpoints appropriate to the aim of the study; (5) Unbiased assessment of the study endpoint; (6) Follow-up period appropriate to the aim of the study; (7) Loss to follow-up less than 5%; (8) Prospective calculation of the study size; (9) An adequate control group; (10) Contemporary groups; (11) Baseline equivalence of groups; (12) Adequate statistical analyses.

The consensus was reached by the two reviewers (A.A./G.V.) when there was the difference in opinion on an item. If no consensus was reached, the independent opinion of a third reviewer was decisive (Y.S.). Summaries of the RoB within a study were produced by adhering to the Higgins et al approach. 30 The quality assessments of the studies included in this systematic review and meta-analysis are given in Tables 3 , 4 , 5 , 6 , 7 .

**Table 6:** Meta-analysis

Study	Experiment ( *N* )	Control ( *N* )	SMD	SE	95% CI	*t*	*p*	Weight (%)	
								Fixed	Random
Pavlin et al (2015)	23	23	0.524	0.295	–0.0704 to 1.118			34.16	27.42
Leethanakul et al (2016)	15	15	1.47	0.403	0.645 to 2.296			18.31	23.24
Miles et al (2018)	20	20	0.102	0.31	–0.525 to 0.730			30.89	26.83
Taha et al (2020)	11	10	–0.328	0.422	–1.212 to 0.557			16.65	22.5
Total (fixed effects)	69	68	0.425	0.172	0.0845 to 0.766	2.468	0.015	100	100
Total (random effects)	69	68	0.439	0.339	–0.231 to 1.109	1.297	0.197	100	100

Abbreviations: CI, confidence interval; SE, standard error; SMD, standardized mean difference.

**Table 7:** Publication bias analysis

Egger's test	
Intercept	1.1136
95% CI	–31.6035 to 33.8306
Significance level	*p* = 0.8970
Begg's test	
Kendall's Tau	–0.3333
Significance level	*p* = 0.4969

Abbreviation: CI, confidence interval.

### Summary Measures, Approach to Synthesis, and Planned Methods of Analysis

The data were grouped and classified according to the type of intervention into two broad categories: experimental group (vibration therapy) and the control group. The majority of the studies evaluated the rate of canine movement as an objective measure and was selected for the meta-analysis. Out of four selected studies in the meta-analysis, one study has data in form of mean and 95% confidence interval (CI), one had provided rate of canine movement for three consecutive months, and one had rate of canine movement for right and left side, individually. All this data was converted to mean and standard deviation for the rate of canine movement (mm/month) by calculating variance and using a statistical formula. Four studies were included in the meta-analysis for the correction of the mandibular anterior irregularity, and two studies were included in the subgroup analysis.

The heterogeneity among studies in each subgroup was evaluated by *I* ^2^ and *Q* statistic and the between-group comparison was conducted in a mixed-effects meta-regression model assuming random study effects of intervention type (VF or controls). We used RevMan 5 software (Copenhagen, Denmark) to make forest plots and meta-analyses. A *p* -value smaller than 0.05 was deemed to be statistically significant.

## Results

### Study Selection

**Fig. 1:**
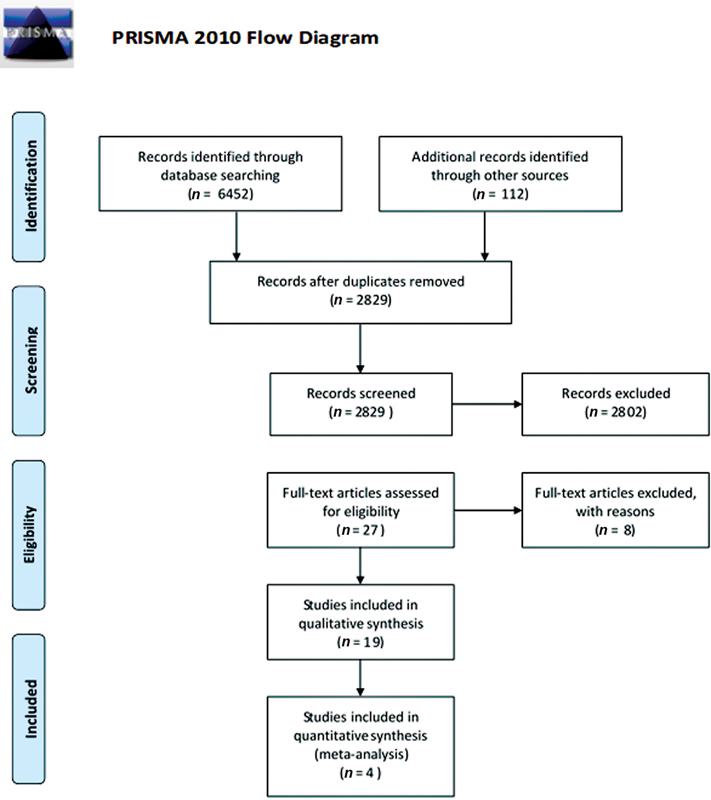
The flow of information through the different phases of a systematic review.

The search strategy yielded a total of 6,452 results, and the records after the removal of the duplicates (2,829) were screened. Seventeen studies were identified for inclusion in the systematic review ( Tables 1 and 2 ). The search strategy and the exclusion of the studies are mentioned in the PRISMA flowchart ( Fig. 1 ).

### Study Characteristics

Out of 19 studies, 6 14 21 24 25 35 36 were animal studies and 13 37 38 39 40 41 42 43 44 45 46 47 48 49 were human studies. The details about population, intervention (vibration parameters), observation, results, and type of study for both animal and human studies are summarized in Tables 1 and 2 , respectively. The RoB assessment was done using the SYRCLE index and ARRIVE criteria for animal studies ( Tables 3 and 4 ). 32 33 Most common missing aspects in the animal studies were missing information about blinding and randomization. For the human studies, a RoB assessment was conducted using the MINORS index. 34 All the studies scored more than 14 on a 24-point scale. The details about the RoB assessment are described in Table 5 .

The applications of VFs in conjunction with orthodontic treatment to accelerate tooth movement have been evaluated through animal studies with mostly positive outcomes. 14 24 25 35 Various levels of VF stimulations were adopted. The rates of tooth movement were different. Table 1 summarized the stimulation levels of these animal studies and the corresponding effects on the tooth. Out of six animal studies, four 14 24 25 36 were conducted on rats (two Wistar 14 24 and two - Sprague Dawley 25 36 ), one on Albino rabbits, 35 and one on CD1 mice. 21 Five 14 21 24 25 35 36 out of six studies evaluated maxillary first molar movement, and one study 35 assessed mandibular incisors ( Table 1 ).

Out of 13 human studies, 8 studies 38 39 40 42 44 45 46 47 48 49 were RCTs, 2 41 43 were split-mouth RCTs, and 1 37 was prospective studies without a control group. Nine studies used AcceleDent as an intervention tool, 37 39 40 42 44 46 47 48 49 one study used VPros, 45 one used tooth masseuse, 38 one was with an electric toothbrush, 41 and one study used a single tooth vibration device. 43 Only one study 41 used thrice a day for VFs and rest of all studies used daily VF application. Six studies 37 39 41 43 44 47 observed rate of canine retraction, and five studies 38 40 42 46 49 observed anterior alignments. Eight 38 40 42 44 46 47 48 49 out of 13 human studies found no significant difference in tooth movement with the VFs ( Table 2 ).

### Rate of Canine Movement

**Fig. 2:**
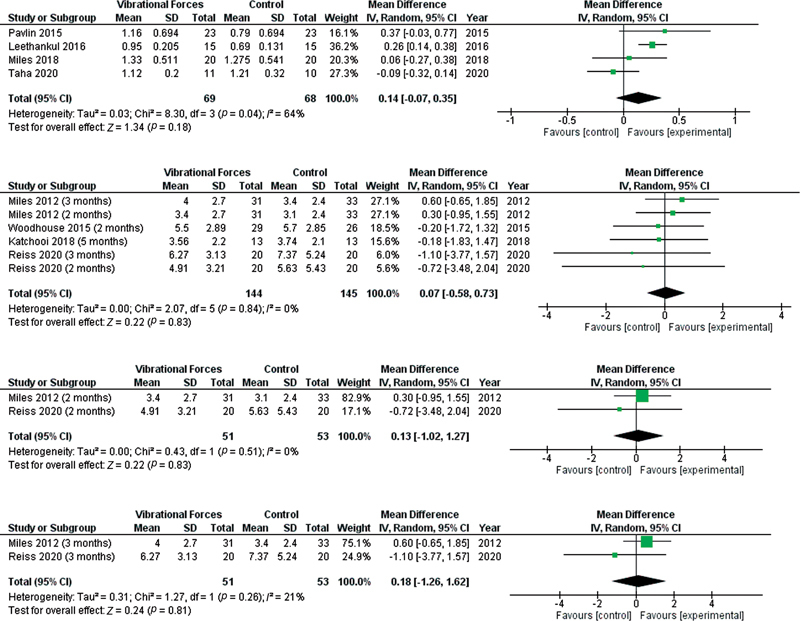
Forest plots showing the comparison of the rate of canine movement (mm/month) between vibrational force and conventional orthodontic treatment.

Pavlin et al 39 reported significant difference in the rate of canine movement between VF group: 1.16 ± 0.694 mm versus control group: 0.79 ± 0.694 mm (mean difference: 0.37 mm, 95% CI: –0.03 to 0.77). Leethanakul et al 41 showed significant difference in the rate of canine retraction between the VF group (0.95 ± 0.205 mm) versus control group (0.69 ± 0.131 mm) (mean difference: 0.26 mm, 95% CI: 0.14–0.38). Miles et al 44 reported no significant difference between the VF group (1.33 ± 0.511 mm) versus control group (1.28 ± 0.541 mm) (mean difference: 0.06 mm, 95% CI: –0.27 to 0.38). Finally, Taha et al 48 observed no significant difference between the VF group (1.12 ± 0.2 mm) versus control group (1.21 ± 0.32 mm) (mean difference: –0.09 mm, 95% CI: –0.32 to 0.14) ( Fig. 2 ).

### Correction of the Mandibular Irregularity Index

No significant difference was found in the overall analysis among the studies (standardized mean difference [SMD]: 0.00 mm, 95% CI: –0.23 to 0.23). Subgroup analysis for 2 months (SMD: 0.01 mm, 95% CI: –0.37 to 0.40) and 3 months (SMD: 0.03 mm, 95% CI: –0.44 to 0.50) did not show a significant difference in the irregularity index between VF and control groups ( Fig. 2 ).

The heterogeneity analysis results were quite sensitive to the sample size, that is, the number of studies. By the rule of thumb, a *Q* value > 25 or *I* ^2^ > 75% implies considerable heterogeneity. *I* ^2^ was 71% and the *Q* value was 10.32 showing significant heterogeneity ( *p* = 0.02). Considering significant heterogeneity, we reported random effect models for the meta-analysis.

### RoB Across Studies

**Fig. 3:**
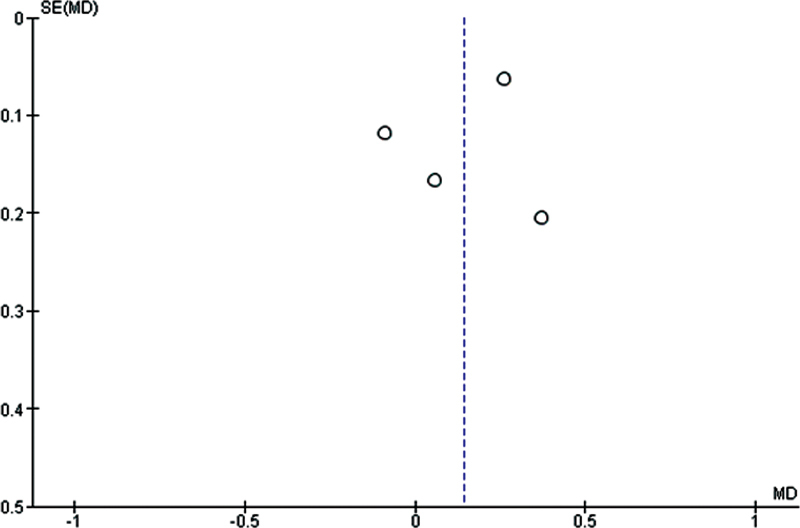
Forest plots showing the comparison of the rate of canine movement (mm/month) between vibrational force and conventional orthodontic treatment.

We evaluated the publication bias for the rate of canine movement (mm/month). The funnel plots with standard error versus SMD after adjusting for intervention type (vibration therapy vs. control) did not show any asymmetric patterns, with no evidence suggesting publication bias ( Fig. 3 ).

## Discussion

The effects of VF on OTM have been studied using both animals and humans. In general, VF has been proven to be effective in accelerating OTM in some the animal studies. The two papers 21 36 that showed negative results had 2 weeks of treatments while others had at least 3 weeks. The duration might be too short to generate a quantifiable rate of tooth movement. The increased tooth displacement due to VF is more significant in 4 weeks than in 2 weeks. 25 One of the two studies (Kalajzic et al) 36 had a PL = 40 cN, which was significantly higher than other reported studies. Excessive PL may not help ATM 14 and may damage the PDL tissues because all tissues have limited load bearing capacity.

Other VF parameters that may affect the ATM are the VF, Af, and AD. The VF within the range of 4 to 120 Hz reported from the reviewed literature may generate accelerated OTM effects, although the level of effects may be different. The Af also varied from twice a week to daily. The AD varied from 3 to 20 minutes. One study 14 demonstrated that the AD has negligible effects on the outcomes in a rat study.

The clinical studies that evaluated the effect of vibration on the rate of OTM, resulted in inconsistent conclusions. The VF can only accelerate the tooth if the tooth senses the stimulation within a certain level of intensity, dose. For instance, comparing two clinical studies conducted by Pavlin et al 39 and Taha et al 48 showed different results although they used the same VF, Af, and AD. This shows that the intensity of VF which is represented by the PL cannot be fully controlled in clinical studies and resulted in different PL. The PL needs to be within a range, otherwise will not be effective. Most clinical studies used commercial devices that consist of a vibrational source and a generic mouthpiece that distributes the VF to the teeth. Neither product provides consistent clinical outcome, nor can they target specific teeth.

The current commercial intraoral vibration device has a generic mouthpiece. It delivers VF to teeth through their contacts with the mouthpiece. Finite element analysis of the commercial vibratory device with ideal occlusion showed that the force distribution over the teeth is not even and anterior teeth receive more stimulus than the posterior. 50 The level of the PL also depends on the stiffness of the mouthpiece and the contact condition. 50 The mouthpiece does not guarantee teeth contacts because each patient has a different teeth profile in terms of height and angulation, and the VF distribution depends on the mouthpiece's stiffness, meaning some teeth receive more PL than others even though all teeth are in contact. Therefore, the same device will have different clinical effects if the targeted teeth are not the same. For example, the incisors may have a better response than the posterior teeth because they received more stimulations. Unless perfectly leveled, some teeth may not be in contact with the mouthpiece, resulting in no stimulation. Without sufficient dose and targeted delivery, desired biological responses do not occur. This is likely a primary reason, why current products do not yield consistent clinical outcomes. Consequently, these devices will not be able to selectively stimulate targeted teeth with a controllable level of stimulation. The animal studies have more consistent results than clinical studies because the VF can be better controlled. The VFs in these studies were directly applied to the tooth with controllable VF intensity.

## Conclusion

Although VFs have been shown to increase the rate of OTM in the experimental studies, the outcomes from the clinical studies were inconsistent. The inability to apply desired PL to the targeted teeth may be the main factor. To ensure clinical efficacy, an adequate level of vibrational stimulation needs to be reliably delivered to the targeted tooth.
