# Supplementary material for: Vibrational Force on Accelerating Orthodontic Tooth Movement: A Systematic Review and Meta-Analysis
Source: Eur J Dent. 2022 Dec 13;17(4):951–63. doi: 10.1055/s-0042-1758070 (PMC10756730; doi:10.1055/s-0042-1758070)
Supplement: Supplementary file 1 — Supplementary Material [file 10-1055-s-0042-1758070-s2252121.pdf]

Supplementary File S1: Description of commercially available dental vibrational devices

1. AcceleDent (OrthoAccel Technologies Inc., Houston, Texas, United States): AcceleDent devices have been marketed with the aim of accelerating tooth movement during orthodontic treatment. They have a mouthpiece that applies 30 Hz VFs directly to the teeth. Patients are instructed to use the device for only 20 minutes per day.
2. VPro5 (Propel Orthodontics, Milpitas, California, United States): VPro5 is another commercial product. It also uses a mouthpiece to apply 120 Hz VFs to the teeth for 5 minutes per day.
3. Tooth Masseuse (no longer available): This device is built to reduce the pain from the treatments with soothing vibration. It can be set for "high" or "low" depending on the level of soothe needed.
4. Electronic toothbrush (Oral-B Triumph, OD17; Procter & Gamble, Cincinnati, Ohio, United States): It is an oscillating-rotating electric toothbrush with a specially designed orthodontic brush head, which delivers a 125 Hz VF to the teeth.

 © 2022. The Author(s).
